# Public acceptability of financial incentives to reward pregnant smokers who quit smoking: a United Kingdom–France comparison

**DOI:** 10.1007/s10198-017-0914-6

**Published:** 2017-06-23

**Authors:** Noémi Berlin, Léontine Goldzahl, Linda Bauld, Pat Hoddinott, Ivan Berlin

**Affiliations:** 1 Department of Economics, European University Institute, Villa la Fonte, Via delle Fontanelle, 18, 50014 San Domenico Di Fiesole, Italy; 2 Université Paris-Dauphine, PSL Research University, Paris, France; 3 UK Centre for Tobacco and Alcohol Studies and Institute for Social Marketing, University of Stirling, Stirling, UK; 4 Nursing, Midwifery and Allied Health Professions Research Unit, University of Stirling, Stirling, UK; 5 Department of Pharmacology, Hôpital Pitié-Salpetrière, Faculté de médecine-Université P. & M. Curie, INSERM U1018, Paris, France

**Keywords:** Acceptability, Financial incentives, Smoking behaviour, Pregnant women, I12

## Abstract

A substantial amount of research has been conducted on financial incentives to increase abstinence from smoking among pregnant smokers. If demonstrated to be effective, financial incentives could be proposed as part of health care interventions to help pregnant smokers quit. Public acceptability is important; as such interventions could be publicly funded. Concerns remain about the acceptability of these interventions in the general population. We aimed to assess the acceptability of financial incentives to reward pregnant smokers who stop smoking using a survey conducted in the UK and then subsequently in France, two developed countries with different cultural and social backgrounds. More French than British respondents agreed with financial incentives for rewarding quitting smoking during pregnancy, not smoking after delivery, keeping a smoke-free household, health service payment for meeting target and the maximum amount of the reward. However, fully adjusted models showed significant differences only for the two latter items. More British than French respondents were neutral toward financial incentives. Differences between the representative samples of French and British individuals demonstrate that implementation of financial incentive policies may not be transferable from one country to another.

**Electronic supplementary material:**

The online version of this article (doi:10.1007/s10198-017-0914-6) contains supplementary material, which is available to authorized users.

## Introduction

In 2012 in France, the prevalence of smoking was 18.9% and 13.4% in the second and third trimester, respectively [4]. Smoking during pregnancy in England was characterized by a progressive decline in smoking rate, but still 12% of mothers self-reported smoking at delivery for 2013–14 (Health and Social Care Information Centre [6]. Using financial incentives for smoking cessation is growing. Although studies of financial incentives have had mixed results in different populations, the evidence of effectiveness for smoking cessation in pregnancy seems to be strong. Two subsequent Cochrane reviews found approximately similar efficacy for incentive based interventions when compared with a non-contingent intervention. In Cahill et al. [2], a meta-analysis based on seven trials conducted in the USA and one in the UK, the adjusted OR at longest follow-up (up to 24 weeks post-partum) was 2.73 (95% CI 1.72–4.35; 1295 participants, moderate-quality studies) in favour of financial incentives. Chamberlain et al. [3] reviewed psychosocial interventions and reported a risk ratio of 2.73 (95% CI 1.72–4.35) when comparing financial incentive as a single intervention vs usual care on abstinence in late pregnancy. A review [7] of 6 controlled trials among economically disadvantaged pregnant smokers supported the efficacy of financial incentives to increase smoking abstinence rates antepartum and early postpartum. Three trials provided evidence that financial incentives improve foetal growth, birth weight, and breastfeeding duration—all of which are negatively affected by smoking. Finally, Tappin et al. [13] published the results of a randomized trial of financial incentives for smoking cessation in pregnancy and reported positive outcomes; the financial incentives group had higher abstinence rate than the control group who did not receive incentives: 22.5 vs 8.6%; the relative risk of not smoking at the end of pregnancy was 2.63 (95% CI 1.73–4.01).

These encouraging results have led researchers to examine public perceptions of this type of policy. Promberger et al. [11] studied the acceptability of financial incentives in a large range of health behaviours such as weight loss, adherence to treatment programs, drug addiction and smoking cessation in the US and the UK. Their study revealed disapproval amongst the public of financial incentives to induce changes in health behaviour in both countries. In another study, Promberger et al. [10] used a discrete choice experiment to investigate whether willingness to accept financial incentives for smoking cessation and weight loss was related to effectiveness (financial incentives being said to be effective at a rate ranging from 5 to 40% compared to a standard treatment with efficacy set at 10%) or whether it depended on the type of incentives. They found that public acceptability increased with the financial incentives’ efficacy and that grocery vouchers were preferred to cash or vouchers for luxury items. Age, gender and educational attainment did not have any impact on the level of acceptability. Overweight or obese individuals and daily smokers were more supportive of financial incentives than those who never experienced these health behaviours. Giles et al. [5] used the same methodology to investigate public acceptability of financial incentives for smoking cessation, physical activity, vaccination and screening in the UK and whether acceptability varied according to socio-demographic characteristics. They found that, in most cases, people tended to be indifferent about financial incentives. Younger respondents and men were more likely to support incentives than older respondents and females, except for smoking cessation. Respondents also preferred financial incentives to be universally provided rather than targeting either low-income households or pregnant women. However, findings from these studies are not necessarily directly transferrable to the issue of incentives for smoking cessation among pregnant smokers.

Turning to financial incentives for smoking cessation in pregnancy, Lynagh et al. [9] looked at their reported acceptability amongst 213 Australian pregnant women attending antenatal clinics. A majority of participants (60%) disagreed or strongly disagreed with the idea of paying cash to pregnant women to quit smoking; only a quarter of the participants agreed or strongly agreed with it, and 15% remained undecided. Forty-three percent of the pregnant smokers agreed with providing financial incentives for smoking cessation among pregnant women but only 23% of non-smoking respondents agreed with incentives.

In the UK, a survey of public acceptability of incentives for smoking cessation in pregnancy and for breastfeeding has been completed [8]. Here we focus on the smoking cessation results only. The survey was conducted in a representative sample of the UK population. It found that 40.5% of those interviewed either strongly agreed or agreed, 42.3% either strongly disagreed or disagreed and 17.2% neither agreed nor disagreed with giving shopping vouchers to pregnant women to support them to stop smoking. The survey identified independent predictors for agreeing with financial incentives for smoking cessation in pregnancy: being of childbearing age, 18–44, compared to those aged 65 and over; being a current smoker who had tried to quit compared to a never smoker; social grade E (non-working) compared to social grade AB (upper middle class, middle class occupations); and people from non-white ethnic groups compared to white British. Independent predictors for not agreeing with financial incentives for smoking cessation in pregnancy were: women compared to men, and those with a lower level of education compared to those with a higher level of education. The authors concluded that the British population had mixed views towards the acceptability of giving financial incentives to smoking pregnant women to help them quit.

Following these previous studies in the US, Australia and the UK, a randomized control trial that aims at testing the efficacy and efficiency of financial incentives for French pregnant smokers began in April 2016 [1]. If this on-going randomized trial confirms the efficacy of financial incentives among pregnant smokers in France, incentives might potentially be proposed as part of a care pathway to reward pregnant smokers who stopped smoking. In that case, public acceptability becomes an important issue since health care interventions are publicly funded and public support can aid implementation.

The aim of the current study was to compare the opinion of the French general public on the acceptability of financial incentives to reward pregnant smokers who quit smoking to the opinion of the general public of the United Kingdom.

## Material

### Data collection

#### In the UK

In the UK, Ipsos MORI (https://www.ipsos-mori.com/) used a controlled form of random location sampling to identify 161 geographical sites using a method of quota sampling, which has been independently evaluated [8]. Trained field researchers were asked to interview five people at home from 250 addresses at each site, to obtain a nationally and regionally representative sample of adults aged 18 or over between 22 March 2013 and 15 April 2013 (*N* = 1144). Interlocking quotas were set for age, sex, working status and tenure based on the known profile of Great Britain. Interviews took place in person and interviewers used computer assisted personal interviewing (CAPI). Incentive questions were asked after the demographic questions, but before smoking and breastfeeding status questions. The order for the smoking and breastfeeding questions was randomized to assess framing effects. This made no significant difference to the acceptability of financial incentives for smoking cessation [8].

#### In France

The UK team agreed to give the French team the questionnaire used in their study [8]. The questionnaire was first translated to French then back translated; the back translation was checked for accuracy against the original English version. Both questionnaires can be found in the online Appendices. The British questionnaire included questions about breast-feeding that were not included in the French survey.

The French survey was conducted between the 19th and 24th of January 2015, a few weeks before the French media relayed the results of the UK randomized trial conducted by Tappin et al. [13]. Ipsos France (http://www.ipsos.fr/) used the same methods as in the UK survey to identify a representative sample of 1254 people living in France aged from 18 to 69 years old. The sample was stratified according to quota method on gender, age, region and the agglomeration size in a similar way to the British sample. The only difference in the conduct of the survey between the two countries was that the British survey was conducted face-to-face in the home of respondents, whereas it was conducted over the phone in France, due to funding limitations.

### The survey

The five questions used in the French and the UK surveys asked about agreement and disagreement with the provision of shopping vouchers to women who prove that they have stopped smoking in pregnancy. Acceptability was measured on a five-point Likert scale from strongly disagree to strongly agree, with a neutral option “Neither agree nor disagree” (NAND). The questions were:

*Question 1 [hereafter pregnant women SS-vouchers (stop smoking)]* Do you agree or disagree that shopping vouchers should be provided to women who prove that they have stopped smoking during pregnancy?

Some women start smoking again after the birth of their baby, particularly if their partner or someone at home smokes. Please tell me whether you agree or disagree with each of the following statements.

*Question 2a (hereafter women after birth SS-vouchers)* It is acceptable to provide shopping vouchers to a woman for 2 months after the birth of her baby if she proves that she is still not smoking.

*Question 2b (hereafter smoke-free home after birth-vouchers)* It is acceptable to provide shopping vouchers to a woman for 2 months after the birth of her baby if she never lets anyone smoke in her home.

*Question 3 (hereafter health service payment for meeting SS target)* Do you agree or disagree that local health services should receive additional funding if they reach targets for the number of women who prove that they have stopped smoking during pregnancy?

If the respondent answered “strongly agree”, “tend to agree” or “neither agree nor dis- agree” to Question 1, then she is asked:

*Question 4 (hereafter targeted women)* Do you think that it is acceptable to provide shopping vouchers to women who prove that they have stopped smoking during pregnancy, regardless of their income, or only to women on low incomes?

*Question 5 (hereafter maximum amount)* What is the highest amount of shopping voucher you think it would be acceptable to provide a woman who proves that she has stopped smoking during pregnancy?

The possible answers were £/€2, £/€10, £/€20, £/€40, £/€60, £/€80.

Those questions were asked after the socioeconomic questions which were: age, gender, education level, income level, working status, social grade.

Each survey in UK and in France had an a priori target sample size of 1000 to allow us to estimate proportions to within a 3% margin of error with 95% level of confidence.

Both data sets, from the UK and from France are then pooled together in order to perform the analysis.

## Methods of analysis

Hoddinott [8] raised a limitation of their study on “unknown generalization to other countries”. Hence, our primary interest is to evaluate any significant differences in responses between France and the UK. Every answer to the questions cited above were summarised by mean percentages using bar charts broken down by country, UK or France. Differences in the distribution of those answers between countries were tested with Kolmogorov–Smirnov tests.

We investigated the effect of being from the UK, compared to being from France, on the likelihood of accepting financial incentives for smoking cessation among pregnant women (for Questions 1, 2a, 2b and 3) with a linear probability model (LPM, see equation below). We chose to study the answers of those who expressed an opinion; hence, a net agreement or disagreement. We grouped the strongly agree and agree together as well as the strongly disagree and disagree answers, such that for each question, each outcome was a dummy variable which equals 1 if the respondent agrees with the proposition and zero otherwise.

Our main explanatory variable of interest was the dummy variable UK = 1 if respondents were from the UK, 0 if they were from France.

$$A_{iq} = \alpha_{iq} + \beta_{iq} UK_{iq} + \gamma_{iq} \, X_{iq} + \varepsilon_{iq} ,$$

where *A*_i_ is the dummy variable = 1 if respondents i agree with the proposition *q* = {1, 2a, 2b, 3}, 0 if they do not (NAND excluded); UK_*i*_ is a dummy variable equal to 1 if respondents are from the UK and 0 if from France, and *X*_i_ is the other observed covariates (gender, age, smoking status, social grade, education level and income level). *ε*_*iq*_ is assumed to be normally distributed with a mean of zero and variance *σ* ^2^. Each model was estimated using clustered error terms at the region level because cultural similarities and health care system differences at the scale of the region may induce correlation between unobservable and biased estimates. The answer to question 4 (targeted women) was also studied with a LPM on the dummy variable equal to 1 if the respondent thinks it is best to target low-income women, 0 otherwise. For Question 5 (maximum amount), an ordered logit was implemented. We then studied whether the effect of socioeconomic variables differed with the country of origin using interaction terms. In this case, each interaction effect was run in a different model, fully controlled and using clustered error terms at the region level.

In order to check the robustness of the results when the NANDs are included, we performed the analysis with an ordered logit on the whole sample for every question. This does not change the main results we describe in the paper. We have also run a multinomial logit for which we set the net disagreement as the reference. The results showed by the bar charts hold when variables of control were also included. The multinomial logit regressions, for which the estimates are less straightforward to interpret, did not provide us with more information than those we present in the current paper. Hence those tables are not reported here, but are available upon request.

## Results

### Sample characteristics

Table 1 shows the characteristics of the UK (*N* = 1144) and the French (*N* = 1254) sample. Sample distribution in terms of social grade, education levels and income are statistically different. More British than French respondents refused to answer the questions about their income (40 vs 12.6%) and smoking status (4.5 vs 0.7%).

**Table 1:** Descriptive statistics of the French and the UK samples

	France	UK
Age groups, K–S test *p* value = 0.17		
18–24	10.8%	14.9%
25–34	15,5%	15.3%
35–44	18.9%	15.8%
45–54	15.9%	13.9%
55–59	9.5%	6.3%
60–64	7.7%	8.2%
65+	21.7%	25.6%
Female, *p* value <0.01	43,8%	52.8%
Missing	0.2%	0.0%
Smoking status, K–S test *p* value = 0.06		
Never smoker	44.7%	50.1%
Former smoker	29.4%	24.6%
Current (tried quitting)	16.7%	15.3%
Current (not tried quitting)	8.5%	5.5%
Refused to answer	0.7%	4.5%
Has children, *p* value <0.01		
Yes	70.9%	64.9%
Social grades^a^, K–S test *p* value <0.01		
AB (upper middle class and middle class/executive)	24.2%	20.9%
C1 (lower middle class/employee)	16.7%	12.0%
C2 (skilled working class/farmer, craftsmen)	4.6%	32.3%
D (working class/workers)	12.9%	20.6%
E (non-working, retired, student)	41.6%	14.2%
Education level, K–S test *p* value <0.01		
University degree	41.53%	25.79%
A-level/Bac	17.57%	16.87%
Vocational education/CAP	27.88%	10.58%
No formal qualification-GCSE/BEPC	11.58	36.54%
Other, still studying, don’t know	1.44%	10.23%
Income in quintiles, K–S test *p* value <0.01		
2501–4500€/£per month	22.5%	11.9%
1501–2500€/£per month	24.4%	12.8%
900–1500€/£per month	18.8%	10.0%
<900€/£per month	12.1%	11.2%
Refused to answer	12.6%	40.1%
Observations (*N*)	1254	1144

The *p* value of Kolmogorov–Smirnov distribution tests (K–S test) of variables between countries and *p* value from test on the equality of proportions are reported. 0.2% of the French sample (*N* = 2) refused to reveal their gender: they were systematically removed from the econometric analysis unless specified otherwise

*CAP* Certificat d’aptitude professionnelle, *Bac* Baccalauréat, *BEPC* Brevet d’Etudes du Premier Cycle, *GCSE* General Certificate of Secondary Education

^a^Social grades are classified in a different manner in France and in the UK so we tried to group them in a consistent way

### Acceptability of financial incentives

#### Proportions by country

**Fig. 1:**
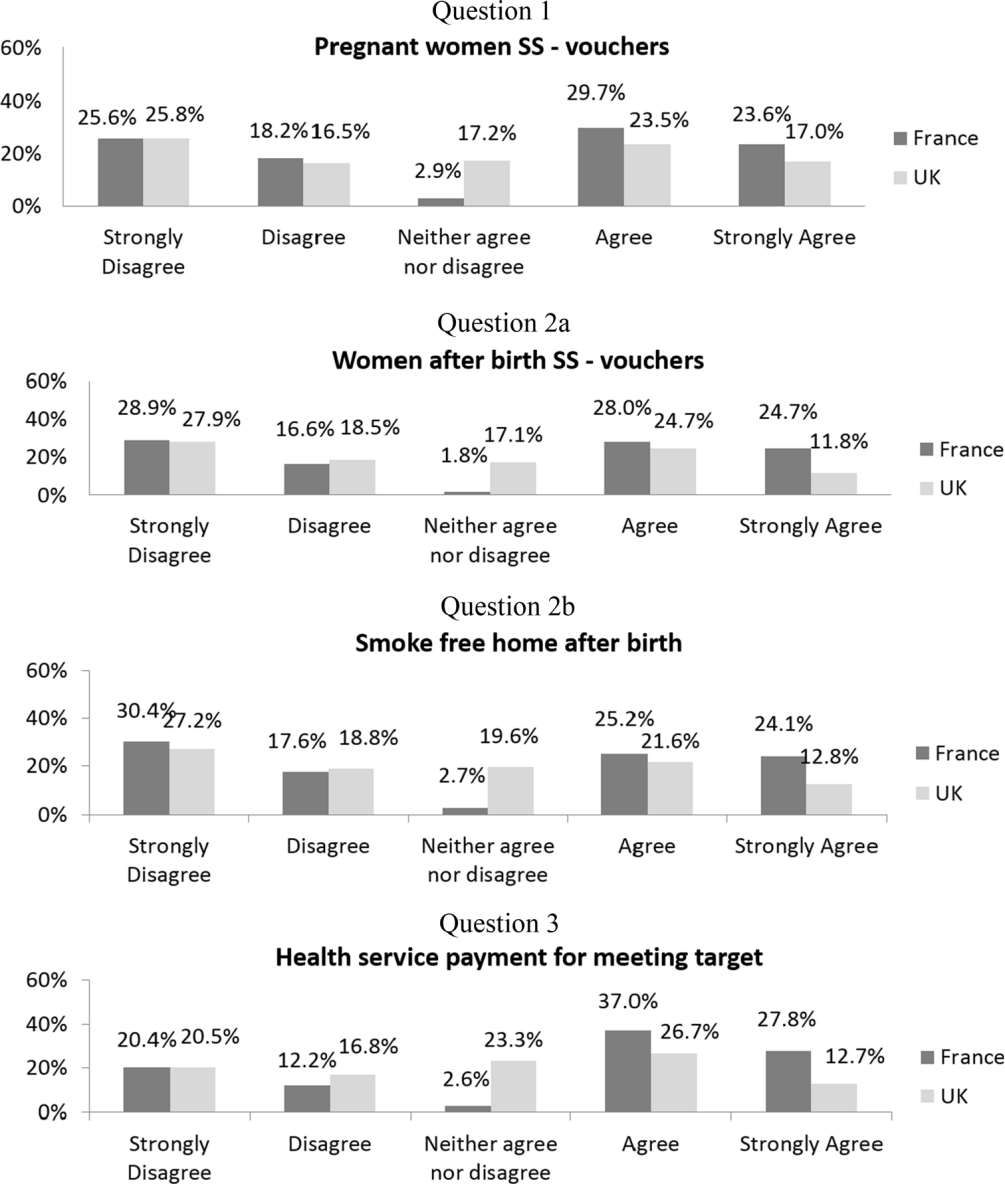
Proportions of responses by country

**Fig. 2:**
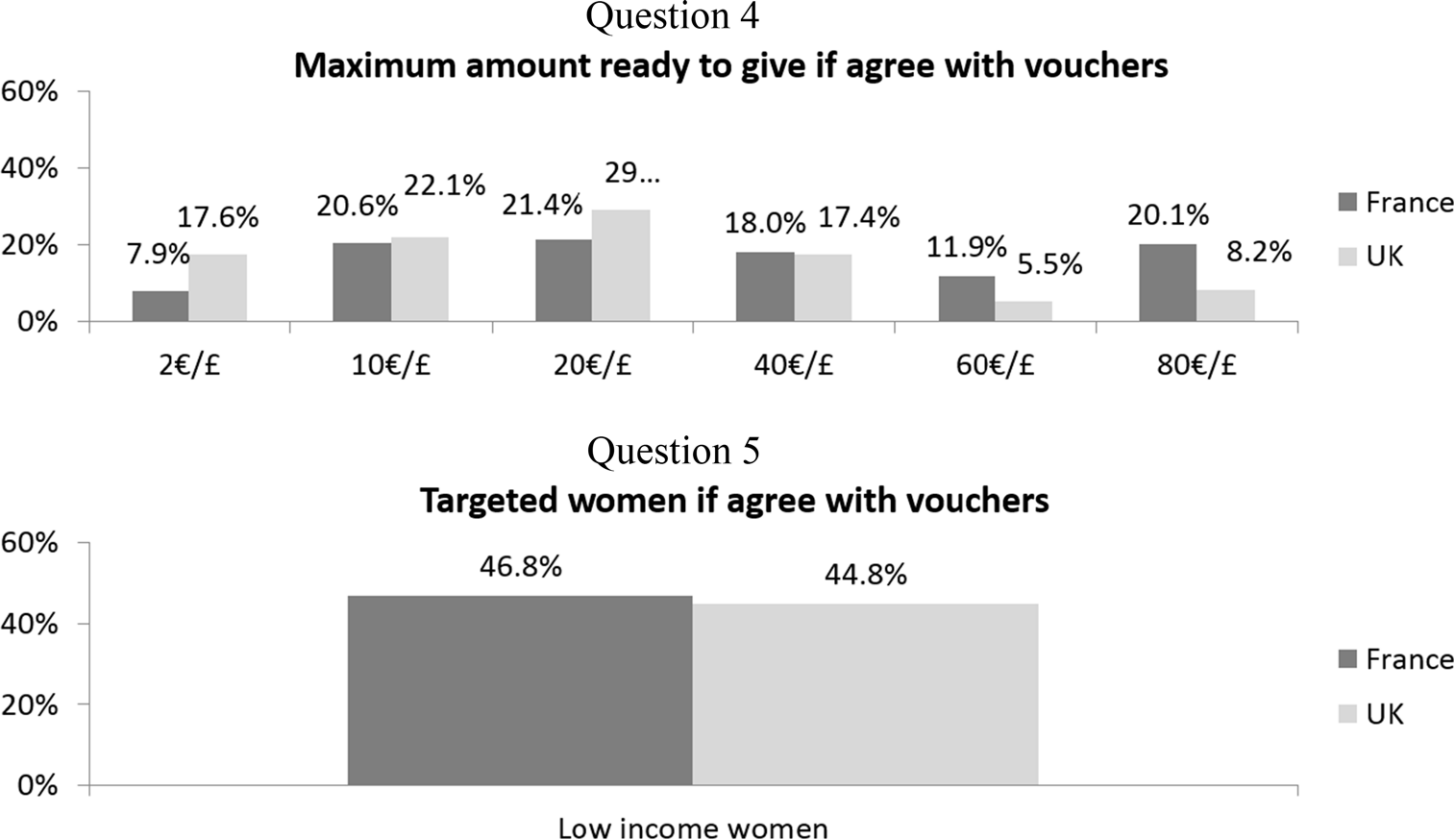
Proportions of responses by country among those who agree and neither agree nor disagree with financial incentives

Figures 1 and 2 show the proportion of answers for the different questions asked of French and UK respondents. For all questions, British respondents tended to be more neutral and less likely to agree than the French. For the maximal amount of financial incentive to give to the pregnant women (Fig. 2, question 4), British answers were more skewed towards the lower amounts and the French answers towards the higher amounts (Kolmogorov–Smirnov distribution test yields a *p* value <0.01). About 45% of both French and British respondents thought that financial incentives should only be offered to low-income women rather than to all pregnant smokers. We also see that the same proportions of British respondents agree with providing financial incentives to either pregnant women or health services, while a larger proportion of French respondents agree with providing financial incentives to the health service rather than to pregnant women.

There were large differences for all questions in the proportions of neither agree nor disagree (NAND) (ranging from 1.8 to 2.9% in the French sample and 17.1 to 23.3% in the British sample, all pairwise comparisons yielded a *p* < 0.00001). However, the NAND was excluded from the regression analyses (see “Methods of analysis”). Therefore, we present our binary dependent variable in Table 2 that only includes the agreement (agree and strongly agree) and disagreement (disagree and strongly disagree) responses. For all questions, the French respondents tended to significantly agree more than to disagree, and British respondents disagreed more often with the propositions.

**Table 2:** Proportion of respondents who agree and disagree

	France		UK		*p* value France vs UK*
	*N*	%	*N*	%	
Pregnant women SS-vouchers					
Disagree	548	45.1	484	51.1	<0.001
Agree	668	54.9	463	48.9	
Women after birth SS-vouchers					
Disagree	569	46.3	531	56.0	<0.001
Agree	660	53.7	417	44.0	
Smoke-free home after birth-vouchers					
Disagree	601	49.3	526	57.2	<0.001
Agree	617	50.7	394	42.8	
Health service payment for meeting SS target					
Disagree	408	33.4	426	48.6	<0.001
Agree	812	66.6	451	51.4	

Neither agree nor disagree respondents are excluded. (*p* values from Wilcoxon rank-sum test)

* Threshold for Bonferroni correction: *p* ≤ 0.0125

#### Regression results

##### Country comparisons

Estimates fully controlled for demographics are reported in Table 3. Overall, British respondents were only significantly less likely to be in favour of financial incentives for smoking cessation when asked the questions about health service payment for meeting the target, and the maximum amount they would agree to provide to pregnant smokers. Being a British respondent decreased the probability by 8.2% of being in favour of providing payment to health services if providers met smoking cessation targets in pregnancy, and they were more likely to choose a lower level of payment, compared to the French.

**Table 3:** Individual characteristics associated with acceptability of financial incentives to help pregnant smokers quit smoking

	Pregnant women SS (1)	Women after birth SS (2)	Smoke-free home (3)	Health service payment (4)	Maximum amount (5)	Target low income (6)
UK	0.005 (0.037)	−0.048 (0.038)	−0.026 (0.039)	−0.082** (0.035)	−0.483*** (0.169)	−0.059 (0.053)
Female	−0.077*** (0.021)	−0.076*** (0.021)	−0.037 (0.022)	−0.025 (0.024)	−0.066 (0.101)	−0.019 (0.029)
Age (ref: 18–24)						
25–34	0.011 (0.044)	0.027(0.039)	0.000 (0.049)	−0.031 (0.032)	0.444* (0.246)	−0.020 (0.060)
35–44	0.001 (0.038)	0.012 (0.038)	−0.013 (0.042)	−0.034 (0.045)	0.587** (0.230)	−0.005 (0.053)
45–54	−0.068 (0.041)	−0.023 (0.036)	−0.045 (0.036)	−0.105** (0.041)	0.499** (0.232)	0.031 (0.057)
55–59	−0.104** (0.043)	−0.070* (0.039)	−0.061 (0.045)	−0.069 (0.047)	0.408* (0.221)	0.123* (0.060)
60–64	−0.068 (0.040)	0.031 (0.037)	0.010 (0.039)	−0.147*** (0.037)	0.393 (0.265)	0.041 (0.068)
65+	−0.168*** (0.043)	−0.114*** (0.039)	−0.121*** (0.040)	−0.221*** (0.044)	−0.156 (0.225)	0.139** (0.061)
Smoking status (ref: never smoked)						
Former smoking	−0.009 (0.022)	−0.32 (0.031)	−0.052* (0.028)	−0.021 (0.035)	−0.011 (0.110)	0.092 (0.035)
Current (tried quitting)	0.052* (0.026)	0.034 (0.024)	0.047* (0.027)	0.038 (0.040)	0.368** (0.158)	0.020 (0.047)
Current (did not try quitting)	0.006 (0.036)	0.014 (0.035)	0.040 (0.036)	−0.021 (0.049)	0.056 (0.306)	−0.063 (0.078)
Refused to answer	0.067 (0.084)	0.087 (0.084)	−0.007 (0.077)	0.078 (0.106)	−0.856* (0.491)	−0.070 (0.092)
Education (ref: University degree)						
A level/Bac	−0.079** (0.033)	−0.040 (0.027)	−0.072* (0.039)	−0.082** (0.032)	−0.554*** (0.138)	0.074 (0.045)
Vocational education/CAP	−0.030 (0.035)	−0.027 (0.042)	−0.016 (0.042)	0.006 (0.029)	−0.071 (0.188)	0.078 (0.046)
No formal qualifications-GCSE/BEPC	−0.030 (0.034)	−0.050 (0.050)	−0.030 (0.051)	−0.023 (0.034)	−0.145 (0.165)	0.092 (0.058)
Other, still studying, don’t know	0.038 (0.044)	0.034 (0.055)	0.016 (0.052)	0.043 (0.052)	−0.453* (0.250)	0.031 (0.095)
Has children	0.050* (0.029)	0.032 (0.030)	0.002 (0.026)	0.011 (0.034)	−0.281* (0.151)	0.008 (0.040)
Social grade (ref: A&B/) Executive and intermediary profession						
C1/employee	0.088* (0.042)	0.081** (0.032)	0.090** (0.033)	−0.014 (0.036)	0.135 (0.206)	−0.109* (0.053)
C2/farmer, craftsmen	−0.013 (0.041)	−0.024 (0.043)	−0.033 (0.040)	−0.091* (0.044)	0.071 (0.173)	0.042 (0.047)
D/workers	0.073 (0.043)	0.076* (0.037)	0.077 (0.047)	0.010 (0.038)	0.271 (0.172)	−0.079 (0.058)
E/not working	0.083** (0.039)	0.049 (0.036)	0.047 (0.038)	0.045 (0.032)	0.107 (0.168)	0.002 (0.054)
income (5^th^ quintile)						
1st quintile	0.046 (0.044)	0.099* (0.044)	0.040 (0.041)	0.022 (0.030)	−0.025 (0.221)	0.078 (0.057)
2nd quintile	0.073 (0.050)	0.134*** (0.046)	0.086* (0.043)	0.116*** (0.037)	−0.119 (0.165)	0.020 (0.041)
3rd quintile	0.026 (0.045)	0.046 (0.038)	0.010 (0.035)	0.023 (0.038)	−0.229 (0.192)	0.012 (0.055)
4th quintile	0.024 (0.057)	0.015 (0.054)	0.020 (0.054)	−0.032 (0.037)	−0.135 (0.130)	−0.055 (0.042)
Refused to answer	−0.055 (0.054)	0.016 (0.047)	−0.030 (0.046)	−0.054 (0.033)	−0.096 (0.157)	−0.009 (0.057)
Constant	0.588*** (0.045)	0.597*** (0.055)	0.558*** (0.054)	0.770*** (0.066)	na	0.485*** (0.081)
Observations (N)	2163	2177	2138	2097	1131	1131
*R*-squared	0.042	0.044	0.039	0.071		0.046
Pseudo *R*-squared					0.0237	

Robust standard errors clustered at the region level in parentheses

Columns (1) to (4) and (6) report coefficients from LPM. Column (5) reports the estimates from the ordered logit

NANDs are excluded

*Bac* Baccalauréat, *BEPC* Brevet d’Etudes du Premier Cycle, *GCSE* General Certificate of Secondary Education

* *p* value <0.1, ** *p* value <0.05, *** *p* value <0.001

##### Individual characteristics that influence acceptability of financial incentives

In order to provide a robustness check of the British data [8] we report here the effects of individuals’ characteristics (*X*_*iq*_) on the public acceptability of financial incentives on the whole sample (UK and France) in Table 3. We reproduced the effect of individual characteristics for each question.

- Women were more likely to disagree with providing financial incentives during pregnancy and after birth than men.
- Current smokers who had previously tried to quit were significantly more in favour of providing vouchers to pregnant women if they stopped smoking and if they maintained a smoke-free home (significant at a 10% level) and they were also in favour of providing a higher amount per month than those who had never smoked (significant at a 5% level).
- Older respondents (65+) were less likely to endorse financial incentives for smoking cessation among pregnant women than younger ones (18–24 years old). The effects were quite large, ranging from 11 to 22% and were highly significant, the larger being for providing funding to health services who meet the targets. Respondents aged between 25 and 59 years old, compared to younger ones, were more likely to choose a higher amount of vouchers if pregnant women stopped smoking.
- Those who had completed secondary school in the UK or France were less likely to support incentives for pregnant women who stop smoking, smoke-free home after birth, and health service payments for meeting target than those who had been educated up to the undergraduate level. These effects ranged between 7.2 and 8.2%. Moreover, they were also less likely to be in favour of providing higher value vouchers to pregnant smokers.
- Being employed or belonging to social grade C1 (lower middle class) compared to higher social grades increased the likelihood of being in favour of financial incentives by 8–11%, except when they targeted the funding of health services. They were also in favour of providing financial incentives universally rather than targeting low-income women.
- Respondents who did not work (social grade E, such as retirees or unemployed individuals) were more supportive of providing vouchers to pregnant women for smoking cessation than those with social grades A or B.

Additional results suggest that former smokers were less supportive than never smokers when asked about the acceptability of providing incentives to women for maintaining a smoke-free home after birth. Also, respondents who had children were slightly more supportive of financial incentives for smoking cessation. However, they favoured lower value vouchers than respondents without children.

Respondents belonging to the 2nd quintile of income tended to agree more with providing vouchers to encourage smoking cessation after birth (women after birth SS), to keep a smoke-free home and payment to health services than those at the top of the income distribution. The effects ranged from 8.6 to 13.4%.

#### Country by individual characteristic interactions

**Fig. 3:**
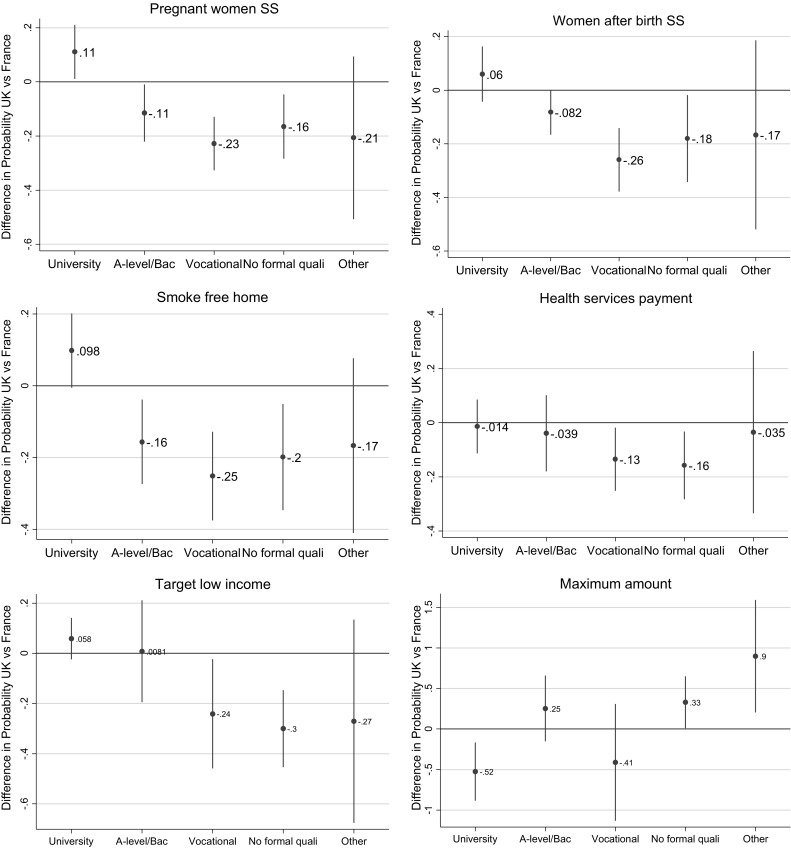
Difference in the probability of accepting financial incentives for smoking cessation among pregnant smokers by level of education: UK vs France. The ordinate axis is the difference in probability of agreeing with financial incentives for British respondents compared to the French ones. A positive difference means a higher probability in the UK

Supplementary Tables 1.a and 1.b report the detailed results of the country by individual characteristics interactions. The main interactions results are depicted in Fig. 3.

Firstly, the effects of education levels differ according to the country (Fig. 3). Compared to those who hold a university degree, British respondents who had a lower educational level (A-level/Bac, vocational studies, no formal qualifications) were significantly less in favour of financial incentives than their French counterparts, for cessation during pregnancy or after birth, smoke-free home after birth, health services payments and targeting low-income women. Among those who had a university level education, British respondents compared to French were more likely to support financial incentives for smoking cessation but chose lower value vouchers.

Secondly, the effect of smoking status differed by country. The French former smokers compared to those who never smoked, were willing to provide higher voucher amounts than their British counterparts (Supplementary Table 1.a).

Thirdly, the effect of social grade differed by country. Not working (grade E) British respondents were less likely to agree with providing vouchers to pregnant women for smoking cessation before and after birth compared to the non-working French respondents (Supplementary Table 1.b).

Lastly, British respondents who had children were more in favour of targeting low-income women compared to French respondents with children (Supplementary Table 1.b).

## Discussion

The aim of this study was to compare the opinions of the French and British general public about the acceptability of financial incentives to reward pregnant smokers who quit smoking. We hypothesised that the acceptability of financial incentives for rewarding pregnant smokers who stopped smoking may be different between countries even if they have similar socioeconomic characteristics, such as the UK and France.

Our data showed that the French population sample was more likely to agree with using financial incentive policies for rewarding smoking cessation during pregnancy, not smoking after delivery, keeping a smoke-free household, health service payment for meeting target and the maximum amount of the reward than the British sample. However, fully adjusted models showed significant differences only for the two latter items. The higher acceptability amongst the French, compared to the British, was mainly driven by individuals with lower levels of education or those people with no employment.

The findings from the combined 2-country sample confirmed previous results of the British survey regarding the association of individual characteristics with agreeing or disagreeing with financial incentives to reward pregnant smokers who quit. Men, younger individuals, low-income respondents, those of lower social grades, current smokers who had tried to quit smoking, and those who had reached tertiary education were more likely to agree with providing financial incentives for smoking cessation among pregnant women. Surprisingly, in both the British and the merged (French and British) samples, women and more affluent respondents were less likely to be in favour of rewarding pregnant women with financial incentives for cessation.

Analysis of the country by individual characteristics interactions showed differential effects at similar levels of socioeconomics, age and education. British respondents were less likely than the French to be in favour of financial incentives for smoking cessation when asked questions about health services payments for meeting targets and the maximum amount they would agree to provide to pregnant smokers. Trialling financial incentives for health services could be considered as a complementary study. This would be more meaningful in France where public acceptability seems to be greater for providing financial incentives for health services than for pregnant women.

As reported in the Methods section, we have also analysed the data while including NAND responses. This sensitivity analysis showed the same results. British respondents were significantly more likely to answer NAND for all questions (see Fig. 1. “Neither agree nor disagree”; all *p* values are < 0.0001), rather than net agreement or disagreement when compared to the French. Overall this means that the British were more likely to be neutral and the French were more likely to declare a net opinion.

*Strengths* Both surveys were conducted by Ipsos MORI using very rigorous quota sampling to obtain nationally representative samples. The UK questionnaire was translated and back translated in order to create a valid questionnaire in French. This reinforced the quality of the comparison. It is also the first survey run in France on the acceptability of financial incentives for behaviour change policy and the first attempt to assess generalizability across countries with similar socio-demographic profiles.

*Limitations* The British survey was conducted face-to-face using computer assisted data collection methods while the French research was a telephone survey, due to limited funds. Could the observed between country differences be due or partially due to this difference in data collection? Szolnoki and Hoffmann [12] reviewed and tested these two methods and concluded that both methods yielded similar results. Also, studies by Hoddinott [8] and Promberger [11] were based on face-to-face and online surveys, respectively, and found comparative trends. Moreover, although British respondents were interviewed face-to-face which should intuitively imply less neutral responses, they were more likely, for every question, to choose the neutral answer compared to their French counterparts. Hence, it is likely that the different ways of collecting data did not account for the observed between-country differences.

We had to make arbitrary decisions as to the categories of social grade in order to fit the specifications of this variable in each country. Consequently, some UK social grades may not always correspond to those in France.

When asking the question about the maximum amount of financial incentives acceptable to reward pregnant smokers who stopped smoking, the displayed amounts were the same in both countries (2 € per month/£2  per month). At the time of writing these two currencies are not dissimilar in value, but the exchange rate between both currencies at the time the survey took place was slightly different.

Some (but not all) surveys analyse data according to the responder’s residence: urban or rural. Although neither of the two surveys asked specifically whether the respondents were living in rural or urban areas, both surveys identified the region the respondents lived in, and analyses used clustered standard errors at the region level.

## Conclusions

Our study identified differences between the public acceptability of financial incentives for smoking cessation in pregnancy between the UK and France. This implies that the implementation of financial incentives policies should not necessarily be based on the transfer of evidence from one country to another, even when the countries are quite similar in terms of socioeconomic characteristics. Public acceptability needs to be investigated in each country before policy implementation, because low public acceptability may impact how interventions like incentives are received. A policy of rewarding smoking cessation amongst pregnant smokers can raise ethical questions, and determining the level of public acceptability in advance could help with negotiating both the ethics and feasibility of such an approach.

## Electronic supplementary material

Below is the link to the electronic supplementary material.

Supplementary material 1 (PDF 36 kb)
