# Supplementary material for: Public acceptability of financial incentives to reward pregnant smokers who quit smoking: a United Kingdom–France comparison
Source: Eur J Health Econ. 2017 Jun 23;19(5):697–708. doi: 10.1007/s10198-017-0914-6 (PMC5948294; doi:10.1007/s10198-017-0914-6)
Supplement: Supplementary file 1 — Supplementary material 1 (PDF 36 kb) [file 10198_2017_914_MOESM1_ESM.pdf]

Appendices

A. Supplementary Tables

Supplementary Table 1.a. Public acceptability determinants with interaction terms for age, education & smoking status

	Pregnant women SS agree (1)	Women after birth SS (2)	Smoke free home (3)	Health service payment (4)	Maximum amount (5)	Target low income (6)
UK	0.015	-0.006	0.032	-0.015	-0.203	-0.080
	(0.052)	(0.043)	(0.057)	(0.064)	(0.220)	(0.101)
25-34	0.024	0.069	0.032	-0.000	0.399	-0.035
	(0.044)	(0.049)	(0.073)	(0.051)	(0.259)	(0.080)
35-44	-0.015	0.009	-0.003	-0.019	0.586***	-0.067
	(0.040)	(0.040)	(0.054)	(0.066)	(0.189)	(0.085)
45-54	-0.030	0.015	-0.021	-0.059	0.540**	0.007
	(0.050)	(0.049)	(0.056)	(0.042)	(0.215)	(0.085)
55-59	-0.076*	-0.030	0.001	-0.003	0.376	0.139*
	(0.044)	(0.045)	(0.042)	(0.070)	(0.237)	(0.072)
60-64	-0.081	0.044	0.059	-0.105*	0.547*	0.059
	(0.049)	(0.045)	(0.055)	(0.058)	(0.271)	(0.105)
65+	-0.179***	-0.095**	-0.080*	-0.175**	0.209	0.175*
	(0.051)	(0.045)	(0.042)	(0.074)	(0.204)	(0.092)
UK X 25-34	-0.022	-0.088	-0.071	-0.065	-0.033	0.016
	(0.083)	(0.066)	(0.089)	(0.063)	(0.338)	(0.124)
UK X 35-44	0.053	0.022	-0.014	-0.019	-0.265	0.132
	(0.053)	(0.047)	(0.073)	(0.076)	(0.280)	(0.134)
UK X 45-54	-0.086	-0.080	-0.050	-0.101	-0.263	0.043
	(0.066)	(0.054)	(0.067)	(0.088)	(0.289)	(0.120)
UK X 55-59	-0.070	-0.093	-0.150*	-0.157	-0.060	-0.077
	(0.087)	(0.079)	(0.085)	(0.094)	(0.315)	(0.119)
UK X 60-64	0.029	-0.023	-0.103	-0.085	-0.396	-0.040
	(0.072)	(0.079)	(0.081)	(0.081)	(0.403)	(0.153)
UK X 65+	0.022	-0.039	-0.080	-0.089	-0.618*	-0.066
	(0.071)	(0.072)	(0.086)	(0.088)	(0.307)	(0.113)
Observations	2,163	2,177	2,138	2,097	1,131	1,131
R-squared	0.044	0.046	0.040	0.073	0.087	0.050
UK	0.013	-0.028	0.010	-0.030	-0.316	-0.104
	(0.050)	(0.052)	(0.051)	(0.047)	(0.185)	(0.065)
Former smoker	-0.000	-0.003	-0.017	0.018	0.198**	-0.042
	(0.025)	(0.029)	(0.027)	(0.042)	(0.089)	(0.045)
Current (tried quitting)	0.056*	0.037	0.074*	0.080*	0.360**	-0.024
	(0.031)	(0.026)	(0.037)	(0.043)	(0.161)	(0.053)
Current (did not try quitting)	-0.002	0.017	0.066	0.033	0.122	-0.088
	(0.043)	(0.043)	(0.048)	(0.072)	(0.296)	(0.108)
Refused to answer	0.289**	0.174	-0.057	-0.039	-1.659***	-0.016
	(0.122)	(0.191)	(0.236)	(0.271)	(0.376)	(0.151)
UK X Previous smoking	-0.021	-0.072	-0.082	-0.095	-0.407**	0.077
	(0.045)	(0.059)	(0.054)	(0.065)	(0.170)	(0.073)
UK X Current (tried quitting)	-0.010	-0.003	-0.061	-0.098	-0.126	0.107
	(0.053)	(0.050)	(0.057)	(0.074)	(0.243)	(0.092)
UK X Current (did not try quitting)	0.028	0.003	-0.068	-0.147*	-0.041	0.064
	(0.076)	(0.080)	(0.078)	(0.083)	(0.412)	(0.135)
UK X refused to answer	-0.269*	-0.112	0.042	0.106	1.191**	-0.052
	(0.154)	(0.211)	(0.252)	(0.290)	(0.521)	(0.189)
Observations	2,163	2,177	2,138	2,097	1,131	1,131
R-squared	0.043	0.045	0.040	0.074	0.088	0.048
UK	0.111**	0.060	0.098*	-0.014	-0.524***	0.058
	(0.048)	(0.050)	(0.050)	(0.048)	(0.173)	(0.040)
A level/Bac	-0.041	-0.017	-0.020	-0.073*	-0.558***	0.063
	(0.034)	(0.037)	(0.047)	(0.041)	(0.140)	(0.058)
Vocational studies/CAP	0.035	0.043	0.052	0.042	0.016	0.141***
	(0.034)	(0.039)	(0.041)	(0.035)	(0.166)	(0.041)
No formal qualifications / GSCE / BEPC	0.047	0.037	0.063	0.061**	-0.282*	0.252***
	(0.046)	(0.058)	(0.052)	(0.026)	(0.160)	(0.069)
Other, still studying, don't know	0.161	0.122	0.093	0.039	-1.067***	0.199
	(0.142)	(0.155)	(0.096)	(0.126)	(0.301)	(0.181)
UK X A level/Bac	-0.116**	-0.083*	-0.157**	-0.040	0.254	0.008
	(0.051)	(0.041)	(0.057)	(0.068)	(0.196)	(0.098)
UK X Vocational studies/CAP	-0.230***	-0.262***	-0.253***	-0.137**	-0.410	-0.241**
	(0.047)	(0.057)	(0.059)	(0.056)	(0.347)	(0.105)
UK X No formal qualifications	-0.166***	-0.181**	-0.200**	-0.159**	0.330**	-0.300***
	(0.057)	(0.078)	(0.071)	(0.060)	(0.154)	(0.074)
UK X Other, still studying	-0.209	-0.169	-0.169	-0.037	0.899**	-0.271
	(0.144)	(0.170)	(0.117)	(0.144)	(0.334)	(0.195)
Observations	2,163	2,177	2,138	2,097	1,131	1,131
R-squared	0.049	0.052	0.047	0.075	0.089	0.061

* p-value< 0.1, ** p-value< 0.05, *** p-value< 0.001
Robust standard errors clustered at the region level in parenthesis.
Columns (1) to (6) report coefficients from LPM.
NANDs are excluded

Supplementary Table 1.b. Public acceptability determinants with interaction terms for social grade and having children

	Pregnant women SS agree (1)	Women after birth SS (2)	Smoke free home (3)	Health service payment (4)	Maximum amount (5)	Target low income (6)
UK	0.067	0.024	0.025	-0.026	-0.674***	-0.080
	(0.042)	(0.043)	(0.043)	(0.055)	(0.138)	(0.078)
C1/Employee	0.076	0.100**	0.097**	-0.007	-0.104	-0.147***
	(0.058)	(0.037)	(0.040)	(0.049)	(0.212)	(0.052)
C2/farmer, craftsmen	-0.008	-0.006	-0.012	-0.094	-0.462	0.014
	(0.065)	(0.062)	(0.087)	(0.090)	(0.385)	(0.093)
D/workers	0.126***	0.120***	0.096**	0.058	0.075	-0.097
	(0.031)	(0.032)	(0.044)	(0.040)	(0.210)	(0.059)
E/ Not working	0.142***	0.099**	0.088**	0.085**	0.037	0.012
	(0.041)	(0.035)	(0.038)	(0.036)	(0.173)	(0.046)
UK X C1/Employee	0.042	-0.054	-0.021	-0.017	0.525*	0.110
	(0.071)	(0.061)	(0.059)	(0.066)	(0.267)	(0.098)
UK X C2/farmer, craftsmen	-0.034	-0.059	-0.050	-0.025	0.715*	0.048
	(0.079)	(0.082)	(0.100)	(0.106)	(0.403)	(0.114)
UK X D/workers	-0.112	-0.104	-0.052	-0.108	0.341	0.047
	(0.072)	(0.071)	(0.085)	(0.068)	(0.259)	(0.107)
UK X E/ Not working	-0.164**	-0.148**	-0.128	-0.121	0.098	-0.035
	(0.061)	(0.058)	(0.075)	(0.073)	(0.246)	(0.122)
Observations	2,163	2,177	2,138	2,097	1,131	1,131
R-squared	0.047	0.046	0.040	0.073	0.087	0.048
UK	-0.030	-0.055	-0.044	-0.092	-0.431**	-0.148**
	(0.058)	(0.056)	(0.054)	(0.054)	(0.188)	(0.064)
Has children	0.027	0.028	-0.009	0.004	-0.221*	-0.045
	(0.035)	(0.042)	(0.036)	(0.047)	(0.126)	(0.051)
UK X Has children	0.051	0.010	0.027	0.015	0.001	0.131**
	(0.042)	(0.045)	(0.051)	(0.053)	(0.202)	(0.059)
Observations	2,163	2,177	2,138	2,097	1,131	1,131
R-squared	0.043	0.044	0.039	0.071	0.082	0.050

* p-value< 0.1, ** p-value< 0.05, *** p-value< 0.001
Robust standard errors clustered at the region level in parenthesis.
Columns (1) to (6) report coefficients from LPM.
NANDs are excluded

B. Questionnaire in English

I would now like to ask you some questions about smoking during pregnancy…

CS01. Stopping smoking in pregnancy benefits the health of the baby and the mother. Research shows that providing shopping vouchers to women who prove that they have stopped smoking during pregnancy increases the number of women who stop. While some people feel that providing vouchers is appropriate, others feel that it is wrong or unfair. Do you agree or disagree that shopping vouchers should be provided to women who prove that they have stopped smoking during pregnancy?

Strongly agree

Tend to agree

Neither agrees nor disagrees

Tend to disagree

Strongly disagree

IF CODE STRONGLY AGREES, TEND TO AGREE OR NEITHER AGREES NOR DIS-AGREES AT CS01, ASK: CS02. What is the highest amount of shopping voucher you think it would be acceptable to provide a woman who proves that she has stopped smoking during pregnancy?

A. 2 per month B. 10 per month C. 20 per month D. 40 per month E. 60 per month F. 80 per month

IF CODE STRONGLY AGREES, TEND TO AGREE OR NEITHER AGREES NOR DIS-AGREES AT CS01, ASK:

CS03. Do you think that it is acceptable to provide shopping vouchers to women who prove that they have stopped smoking during pregnancy, regardless of their income, or only to women on low incomes?

- To all women, regardless of income
- Only to women on low incomes

ASK ALL

CS04. Some women start smoking again after the birth of their baby, particularly if their partner or someone at home smokes. Please tell me whether you agree or disagree with each of the following statements.

Statements: CSO4 1 - It is acceptable to provide shopping vouchers to a woman for two months after the birth of her baby if she proves that she is still not smoking

CSO4 2 - It is acceptable to provide shopping vouchers to a woman for two months after the birth of her baby if she never lets anyone smoke in her home

Precode list:

Strongly agree Tend to agree
Neither agree nor disagree
Tend to disagree
Strongly disagree

CS05. Do you agree or disagree that local health services should receive additional funding if they reach targets for the number of women who prove that they have stopped smoking during pregnancy?

Precode list:

Strongly agree Tend to agree Neither agree nor disagree Tend to disagree

Strongly disagree

CS13. Do you currently smoke or have you ever smoked?
Yes, I currently smoke every day
Yes, I currently smoke, but not every day
Yes, I used to smoke but have quit
No, I have never smoked
I prefer not to answer

IF CODE 1 OR 2 AT CS13:

CS14. Have you ever tried to stop smoking?

Yes

No

C. Questionnaire in French

Je voudrais vous poser des questions sur le fait de fumer pendant une grossesse.

CS01 : Arrêter de fumer pendant la grossesse est bon pour la santé du bébé et de la mère. Des études montrent que donner des bons d'achat à des femmes ayant prouvé qu'elles ont arrêté de fumer pendant leur grossesse, augmente le nombre de femme qui arrêtent de fumer. Alors que certaines personnes ont le sentiment que procurer des bons d'achat est approprié, d'autres pensent que c'est mauvais ou injuste. Êtes-vous d'accord avec le fait de donner des bons d'achat aux femmes pour les récompenser d'avoir arrêté de fumer pendant leur grossesse ?

1. Tout à fait d'accord
2. Plutôt d'accord
3. Sans opinion
4. Plutôt pas d'accord
5. Tout à fait en désaccord

Si réponse Tout à fait d'accord, Plutôt d'accord, Sans opinion pour la question CS01, alors demander:

CS02. Quel montant maximum de bons d'achat pensez-vous qu'il soit acceptable de donner à des femmes ayant prouvé qu'elles ont arrêté de fumer pendant leur grossesse ? (Une seule réponse autorisée)

A. 2 euros par mois
B. 10 euros par mois
C. 20 euros par mois
D. 40 euros par mois
E. 60 euros par mois
F. 80 euros par mois

Si réponse Tout à fait d'accord, Plutôt d'accord, Sans opinion pour la question CS01, alors demander:

CS03. Pensez-vous qu'il serait acceptable de donner des bons d'achat aux femmes ayant prouvé qu'elles ont arrêté de fumer pendant leur grossesse, quel que soit leur revenu, ou uniquement aux femmes qui ont un bas revenu ?

1. A toutes les femmes, quel que soit leur revenu

2. Uniquement aux femmes à bas revenu

Demandez à tous :

CS04. Certaines femmes recommencent à fumer après la naissance de leur bébé, en particulier si leur conjoint ou quelqu'un d'autre fume au domicile. Pourriez-vous s'il vous plait nous indiquer si vous êtes d'accord ou en désaccord avec chacune de ces propositions:

Propositions:

CS04_1

Est-il acceptable de donner des bons d'achat à une femme deux mois après la naissance de son bébé si elle prouve qu'elle ne fume toujours pas.

1. Tout à fait d'accord
2. Plutôt d'accord
3. Sans opinion
4. Plutôt pas d'accord
5. Tout à fait en désaccord

CS04 2Est-il acceptable de donner des bons d'achat à une femme deux mois après la naissance de son bébé si elle ne laisse jamais quelqu'un fumer à son domicile ?

1. Tout à fait d’accord
2. Plutôt d’accord
3. Sans opinion
4. Plutôt pas d’accord
5. Tout à fait en désaccord

CS05. Etes-vous ou non d’accord avec le fait que les services de santé devraient bénéficier de fonds supplémentaires si un certain nombre de femmes arrêtent de fumer pendant leur grossesse ?

1. Tout à fait d’accord
2. Plutôt d’accord
3. Sans opinion
4. Plutôt pas d’accord
5. Tout à fait en désaccord

CS13. Fumez-vous actuellement ou avez-vous déjà fumé ?

1. Oui, je fume tous les jours
2. Oui, je fume, mais pas tous les jours
3. Oui, j’ai fumé, mais j’ai arrêté
4. Non, je n’ai jamais fumé
5. Ne se prononce pas

(Si 1 ou 2 à la question CS13 : demandez CS14)

CS14. Avez-vous déjà essayé d’arrêter de fumer ?

1. Oui
2. Non
